# Bi-layered living cellular constructs promote recruitment and activation of an M2 macrophage phenotype

**DOI:** 10.3389/fimmu.2026.1917691

**Published:** 2026-10-01

**Authors:** Justin T. Avery, Steven M. Zabroski, Hattie E. Hensley, Katrina A. Harmon, Kelly A. Kimmerling, Katie C. Mowry

**Affiliations:** 1 Organogenesis Discovery Center, Birmingham, AL, United States; 2 Organogenesis Translational Research Center, Canton, MA, United States; 3 University of North Carolina at Chapel Hill, Chapel Hill, NC, United States

**Keywords:** anti-inflammatory, bi-layered living cellular construct, cellular and/or tissue-based products, chronic wounds, macrophages, skin substitutes, wound healing

## Abstract

**Background:**

Skin substitutes, also known as Cellular and/or Tissue-based Products (CTPs), and wound dressings often are designed to support the innate wound healing response of the patient. The mechanism of action for these products varies, with some products strictly acting as bacterial barriers while others can alter the wound microenvironment by providing additional extracellular matrix components or modulating the inflammatory response. Bi-layered living cellular constructs (BLCCs) are CTPs that mimic human skin and can heal itself in response to wounding *in vitro*. How could signals from BLCC impact a wound microenvironment?

**Methods:**

Biopsy punches of BLCC were placed atop dermal equivalent layers and cultured for up to 9 days. Outgrowth area was assessed and biopsy + outgrowth was evaluated for genetic regulation shifts. Proteins generated by BLCC and secreted into the media were confirmed and characterized by ELISAs, and this growth media was used as conditioned media to assess the potential recruitment and activation of monocytes/macrophages using THP-1 cells.

**Results:**

Biopsies of BLCC had outgrowth for up to 9 days with histological characterization confirming presence of a mature epithelium. Genetic assessment identified several genes involved in activation and migration of cells, as well as several genes involved in the recruitment and activation of monocyte/macrophages, including CCL7, G-CSF, and GM-CSF. Assessment of spent/conditioned media confirmed presence of these proteins and use of spent/conditioned media as a chemoattractant resulted in monocyte recruitment. Cells activated with PMA and exposed to conditioned media were also found to express an M2-like phenotype, as assessed by flow cytometry.

**Conclusion:**

BLCC wounding promotes monocyte recruitment and subsequent polarization into an M2-like population, a critical immune cell effector for the promotion of a wound healing phenotype.

## Introduction

Wounds can largely be classified as either acute or chronic in nature, with acute wounds progressing through the four phases of wound healing whereas chronic wounds often stall out in the inflammatory phase resultant of comorbidities, persistent inflammation, and/or presence of infection/biofilm (1). Treatment of chronic wounds often involves more advanced treatment modalities such as skin substitutes, also known as Cellular and/or Tissue-based Products (CTPs), which are a class of products that, depending on their classification, can range from protective barriers to products that promote wound healing through various mechanisms (2).

Bi-layered living cellular construct (BLCC, Apligraf^®^, Organogenesis, Canton, MA) is a CTP engineered with human fibroblasts and keratinocytes in a bovine collagen matrix (3, 4). It is the only Premarket Approval approved product for the treatment of both diabetic foot ulcers (DFUs) and venous leg ulcers (VLUs), and also has an extensive publication history investigating the mechanism of action in both preclinical models and patients (5–10). Much like normal human skin, BLCC is oriented such that it has a dermal equivalent (DE) layer consisting of fibroblasts and collagen and an epithelial layer (Epi) with mature human keratinocytes. Fibroblasts and keratinocytes remain viable *in vitro* and display characteristics of regenerating skin (11), even maintaining the ability to mount a wound healing response and self-repair when fenestrated (12, 13).

Previous studies have been performed to assess whether BLCC would function as a skin graft. In preclinical rat models utilizing autologous BLCC generated from rat cells, engraftment of BLCC was demonstrated (3, 14). When using athymic mice as a graft model, human BLCC could engraft (15); however, this is likely due largely in part to the lack of T cells, which are the primary mediators of graft rejection (16). BLCC has been shown not to engraft on humans, with a mild response comparable to conventional split thickness skin grafts (17). Cells recruited in response to application of BLCC may therefore not only prevent the CTP from engrafting but lead to an immunological shift in the local wound microenvironment.

While tissue resident macrophages exist in the skin as front-line responders to infection and damage, monocytes are recruited and polarized into macrophages at different stages of the wound healing response and are critical for mounting both the inflammatory response and subsequent tissue remodeling (18). Monocyte-derived macrophages in wound healing are broadly categorized as inflammatory (M1) or anti-inflammatory (M2). A balance of both types is necessary for wound repair, as M1 macrophages are critical in the early stages of wound healing by helping to manage bioburden and clear dead cells, while the transition to M2 promotes an anti-inflammatory environment conducive for cellular migration and wound healing (19). Stimulation of an M2 phenotype inhibits T cell proliferation, thereby promoting tolerance for skin grafts as well as promoting tissue remodeling and inflammation resolution (20). The hypothesis was that BLCC may generate and/or deliver cytokines that promote an M2-like phenotype, allowing it to last longer on the wound bed and promote a wound healing phenotype.

## Methods

### BLCC wound healing assay

To investigate the immunological impact of BLCC on native wound healing responses, we designed an *in vitro* wound outgrowth model to assess genetic changes and the unique microenvironment generated by BLCC. 4mm biopsy punches of bi-layered living cell constructs (BLCC, Apligraf^®^, Organogenesis) were placed upon a dermal equivalent layer (DE), which comprises the bottom layer of BLCC consisting of human dermal fibroblasts with a matrix of type I collagen (21). Samples are cultured at a liquid air interface with proprietary media designed to promote differentiation and migration of cells, which was changed every 3 days. Units were allowed to epithelialize (outgrowth) for 3, 6 or 9 days. At each timepoint, tissue samples were taken for histology, outgrowth, and RT-PCR assessment while spent feed media containing released factors (conditioned media) were collected for use in cellular assays (recruitment and polarization) and cytokine assessment (**Figure 1**).

**Figure 1:**
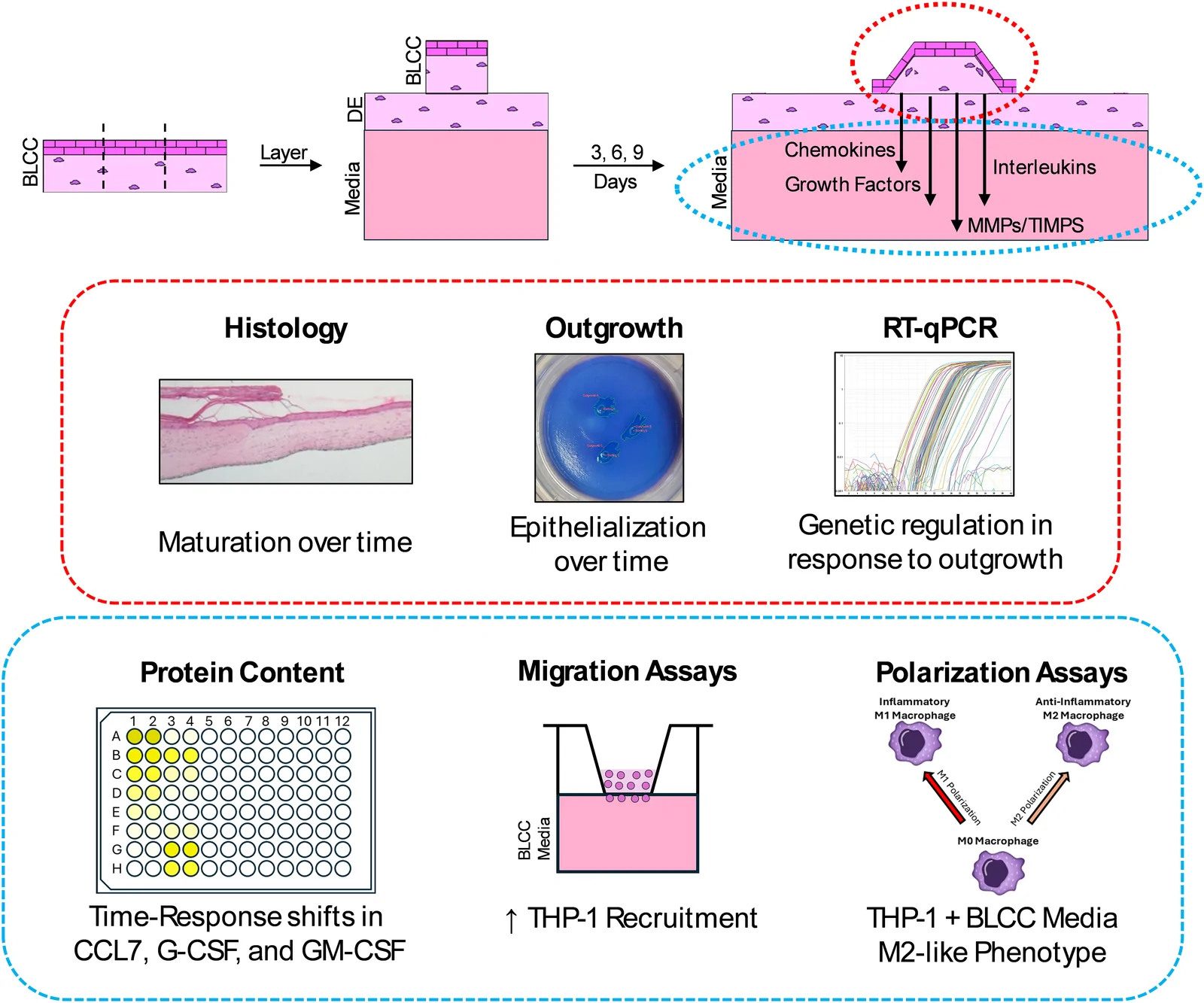
Experimental Model. 4mm biopsy punches of BLCC are placed onto a dermal equivalent layer (DE) and allowed to heal for 3, 6, or 9 days. At each timepoint, designated tissue samples (red circle) and media (blue circle) were collected. Tissue was assessed for histology, outgrowth, and RT-qPCR. Media was assessed for proteins generated by BLCC outgrowth and their impact on monocyte/macrophage (THP-1) recruitment and activation. BLCC, bi-layered living cellular construct; RT-qPCR, real-time quantitative polymerase chain reaction; CCL7, Monocyte Chemotactic Protein-3; G-CSF, Granulocyte Colony-Stimulating Factor; GM-CSF, Granulocyte-Macrophage Colony-Stimulating Factor.

### BLCC characterization with immunofluorescence staining

For immunofluorescence assessment, unwounded BLCC punches were formalin fixed and provided to Inotiv Boulder (Boulder, CO) for processing and imaging. Fixed samples were processed through graded alcohols, cleared with xylene, and infiltrated with paraffin wax. 4 μm tissue sections were taken and immunofluorescence staining was conducted on the Leica Bond RX platform. Antigen retrieval was performed either using heat induced or enzyme digestion with proteinase K. Primary antibodies were incubated with the slide for 30 minutes. Antibody binding was detected using an HRP-conjugated, anti-rabbit secondary polymer, followed by Opal 620 Reagent tyramide signal amplification to allow for immunofluorescence visualization. Spectral DAPI (Akoya Biosciences) was used as a counter stain for all slides and was applied for 10 minutes. Details on antibodies used can be found in **Supplementary Table 1**. Whole slide images were collected using the Vectra Polaris automated quantitative pathology imaging system (Akoya Biosciences) at 20x.

### Nile blue assay

Nile Blue A (Sigma, N-0766) was reconstituted at a concentration of 0.1 mg/mL in water. Outgrowth samples were fully submerged in the 0.01% Nile blue dye overnight at room temperature. After staining, samples were washed in PBS for 30 minutes and pictures were taken. Pictures were taken on a Nikon Eclipse E400 imported into Image-Pro 10 and total outgrowth was calculated. N = 9 for each timepoint assessed.

### Histological assessment of outgrowth

For wound healing samples, biopsies containing the 4mm punch and surrounding outgrowth were processed, sectioned, and stained using standard histological methodology. Hematoxylin and Eosin (H&E) staining was performed to visualize the epithelial tongue as it extended across the DE.

### Reverse transcription-polymerase chain reaction assessment

Three 12mm biopsy punches were taken on day 0, 3, 6, and 9 days so that the 4mm biopsy punch and surrounding outgrowth were captured. Biopsies were stored in RNA*later* (Invitrogen), and RNA was extracted using the RNeasy Fibrous Tissue Mini Kit (Qiagen). RNA was quantified and cDNA was generated using the RT^2^ Human Wound Healing Profiler Array (Qiagen). Fold change was calculated as 2^-ΔΔCt^ and graphed as Log_2_ transformation. Statistically significant genes were input into Search Tool for the Retrieval of Interacting Genes/Proteins (STRING, string-db.org) (10, 22). Genes were clustered with Markov Cluster Algorithm (MCL) inflation rate of 3, and gene ontology (GO) terms, biological process enrichment, and reactome pathway enrichment was performed. N = 3 for each timepoint assessed.

### Enzyme linked immunosorbent assay

Media was changed every 3 days for BLCC healing assays. Spent/conditioned media was assessed for protein generation, specifically C-C Motif Chemokine Ligand 7 (CCL7/MCP-3, DCC700, R&D Systems), Granulocyte Colony-Stimulating Factor (G-CSF, DCS50, R&D Systems), and Granulocyte-Macrophage Colony-Stimulating Factor (GM-CSF, DGM00, R&D Systems) using manufacturer-provided protocols and read at 450 nm with 540 nm correction (Synergy H1 Hybrid reader, BioTek). N = 4–6 for each timepoint/protein assessed.

### THP-1 migration assay

THP-1 cells (ATCC, TIB202) were cultured under manufacturer suggested conditions (ATCC-formulated RPMI-1640 Medium, 10% Fetal Bovine Serum (FBS), 0.05 mM 2-Mercaptoethanol). For migration studies, THP-1 cells were serum starved overnight using low serum media (2.5% FBS). 3 μm transwells (353096, Falcon) were coated with 5 μg/mL fibronectin (RP-43130, Invitrogen) overnight. Transwells were rinsed 3x with PBS to remove unattached fibronectin. 1x10^6^ THP-1 cells were loaded into the top of the transwells. Negative control wells used 2.5% FBS media, positive control wells used 10% FBS media, and test wells included 50% conditioned media with 2.5% FBS from Days 3, 6, and 9 to assess for the ability of BLCC conditioned media to attract monocytes. Cells were allowed to migrate for 24 hours at 37°C in 5% CO_2_ before being counted (Cellometer K2, Revvity) and representative images captured (EVOS M5000, Invitrogen). N = 3 for controls and N = 6 for conditioned media treated wells.

### THP-1 macrophage differentiation

THP-1 cells were seeded at 5x10^5^ cells/well in 6-well plates and allowed to settle for 2 hours. Cells were then stimulated with 150 nM Phorbol 12-myristate 13-acetate (PMA, Sigma-Aldrich, P1585) overnight to differentiate the monocytes into M0 macrophages. The following day, media was refreshed with growth media (10% FBS). The following day, M0 macrophages were polarized into an M1 or M2 phenotype or stimulated with spent/conditioned media from wounded BLCC. For M1 polarization, M0 macrophages were treated with 5 ng/mL lipopolysaccharides (LPS, Sigma-Aldrich, L3024) and 100 μg/mL interferon gamma (IFN-γ, R&D Systems, 285-IF/CF). For M2 polarization, M0 macrophages were treated with 100 μg/mL interleukin-4 (IL-4, R&D Systems, 204-IL/CF) and 100 μg/mL interleukin-13 (IL-13, R&D Systems, 213-ILB). M0 macrophages were also treated with 25% spent/conditioned media from BLCC outgrowth to determine impact of BLCC on macrophage polarization. Cells were assessed at 24 and 48 hours post stimulation by flow cytometry for polarization to an M1 or M2-like phenotype.

### Flow cytometry

Differentiated macrophages were gently removed from 6-well plates using TrypLE Express (Gibco, 12604013). Fc receptor (FcR) blocked cells (Human TruStain FcX™, BioLegend, Cat 422302) and live cells were stained and labeled (LIVE/DEAD Fixable Aqua, Invitrogen, L34957). To assess M1 polarization, cells were stained for CD80 (PE/Cy7 αHuman CD80, BioLegend, Clone 2D10) and CD86 (AF488 αHuman CD86, BioLegend, Clone IT2.2). To assess for M2 polarization, cells were stained for CD163 (PE αHuman CD163, BioLegend, Clone GHI/61) and CD206 (AF647 αHuman CD206, BioLegend, Clone 15-2). Cells were fixed using 4% paraformaldehyde (PFA), acquired on a BD LSRFortessa™, and analyzed using FlowJo™ (version 10.10.0). 2 independent experiments, N = 2 for controls, N = 4 for conditioned media treated samples.

### Statistics

For experiments with multiple groups at a single time point, a one-way ANOVA was performed. For experiments with multiple groups and timepoints, a two-way ANOVA was performed with * p ≤ 0.05. All statistics were performed in GraphPad Prism (version 11.0.0).

## Results

### Characterization of BLCC

BLCC was characterized by immunofluorescence staining for its expression of its extracellular matrix (ECM) components and presence of growth factors (**Figure 2A**). BLCC had a stratified epidermal layer demonstrated by a proliferative phenotype within the basal and suprabasal layers of the epidermis, characterized by Ki67-positive staining. Expression of K19 demonstrated the activation of keratinocytes in the epidermis along with high level expression of VEGF, HGF, and IGF-1 observed. Activated fibroblasts in the dermal layer produced TGF-β as well as other ECM proteins like collagen I, III, IV, and fibronectin. Expression of laminin between the layers highlights the presence of a basement membrane, mimicking native skin architecture. These findings highlight the presence of living active cellular components and ECM proteins in BLCC.

**Figure 2:**
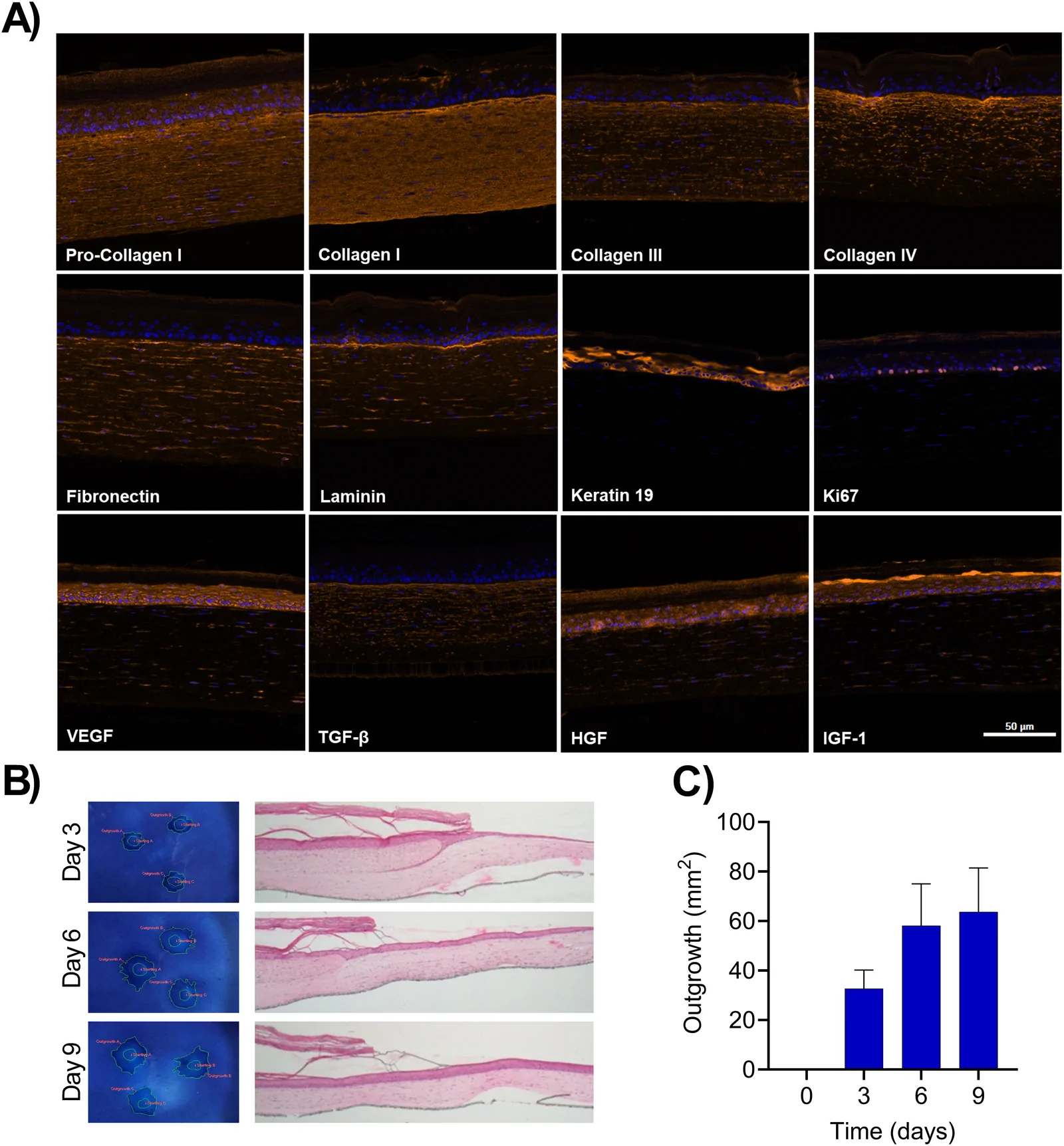
BLCC re-epithelializes in response to wounding. **(A)** Immunofluorescence characterization of BLCC. Scale bar = 50 μm. **(B)** Representative images of histology and Nile Blue staining highlighting outgrowth of BLCC. N = 9 assessed at each timepoint. **(C)** Quantification of total outgrowth area over time; data presented as means ± standard deviations. BLCC, bi-layered living cellular construct; mm, millimeter.

### BLCC re-epithelializes in response to wounding

H&E assessment of wounded BLCCs found early phase epithelialization extending both away from the biopsy as well as undermining the biopsy itself, indicating rapid early phase proliferation and expansion of keratinocytes; by days 6 and 9, the epithelial layer is thicker and predominantly extends away from the biopsy, with little to no undermined epithelialization observable (**Figure 2B**). BLCC had rapid epithelial outgrowth from the wounding site, with an average of 33mm of outgrowth 3 days after wounding, 58mm after 6 days, and starting to plateau by day 9 at 64mm (**Figure 2C**).

### BLCC outgrowth wound healing promotes anti-inflammatory genetic response

BLCC outgrowth resulted in up- or down-regulation of wound healing specific genes and are summarized as a heat map (**Figure 3A**). To determine which genetic regulation shifts were both physiologically and statistically relevant, volcano plots with limits at 2-fold change and -10log(p value) of 1.30 (corresponding to a p value of 0.05) were selected, highlighting several genes of interest across a range of biological pathways that were both up- or down-regulated (**Figure 3B**). Genes involved in the epithelial-mesenchymal transition (EMT) process were differentially regulated during the wound healing process (**Figure 4A**). A reduction of CDH1 (E-Cadherin) and increased expression of genes that encode the collagen integrin α2β1 as well as collagenases and gelatinases (matrix metalloproteinase 1 (MMP1), MMP2, and MMP9) highlight some of the genes involved in the complex EMT process (**Figure 4B**). Statistically relevant genes were subjected to gene ontology (GO) assessment and subsequent biological process and reactome pathway enrichment assessment over the course of the study (**Figure 4C**). Much like wounds in the proliferative or remodeling phases, biological processes and reactome pathways that were enriched included ECM remodeling such as activation of matrix metalloproteinases, degradation of the extracellular matrix, and collagen degradation. Cell chemotaxis was enriched at all time points, which is in line with the continual outgrowth observed over time. Interestingly, it was also observed that pathways for IL-4, IL-10, and IL-13 signaling were all also enriched, which could all promote a wound microenvironment supportive of an M2 polarization phenotype.

**Figure 3:**
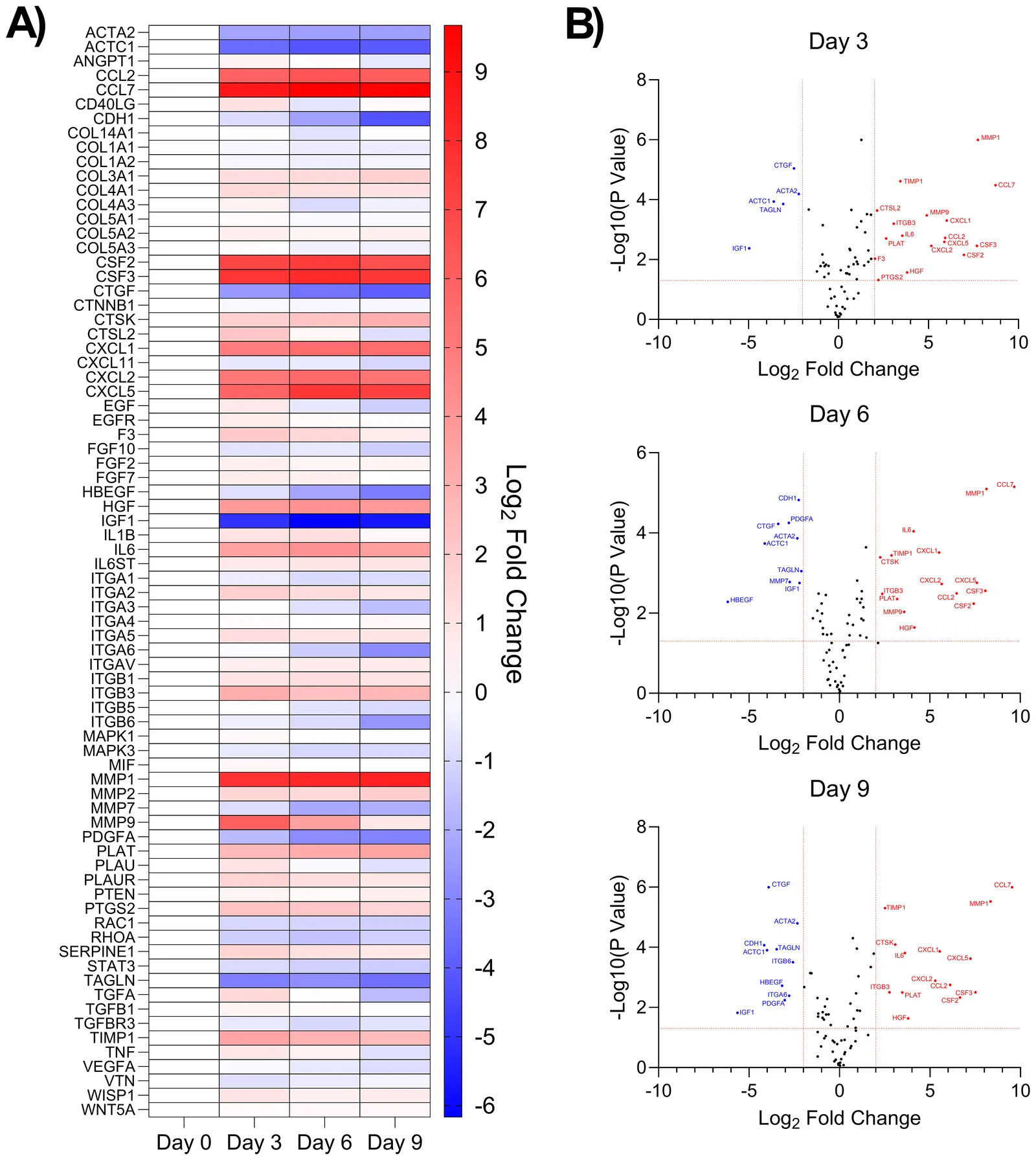
Genetic regulation of BLCC in response to wounding. **(A)** Heatmap showing genes upregulated (red) or downregulated (blue). Log_2_ fold change in expression reported. N = 3 assessed at each timepoint. **(B)** Volcano plots with threshold cutoffs at p values ≤ 0.05 and ± 2 Log_2_ fold changes.

**Figure 4:**
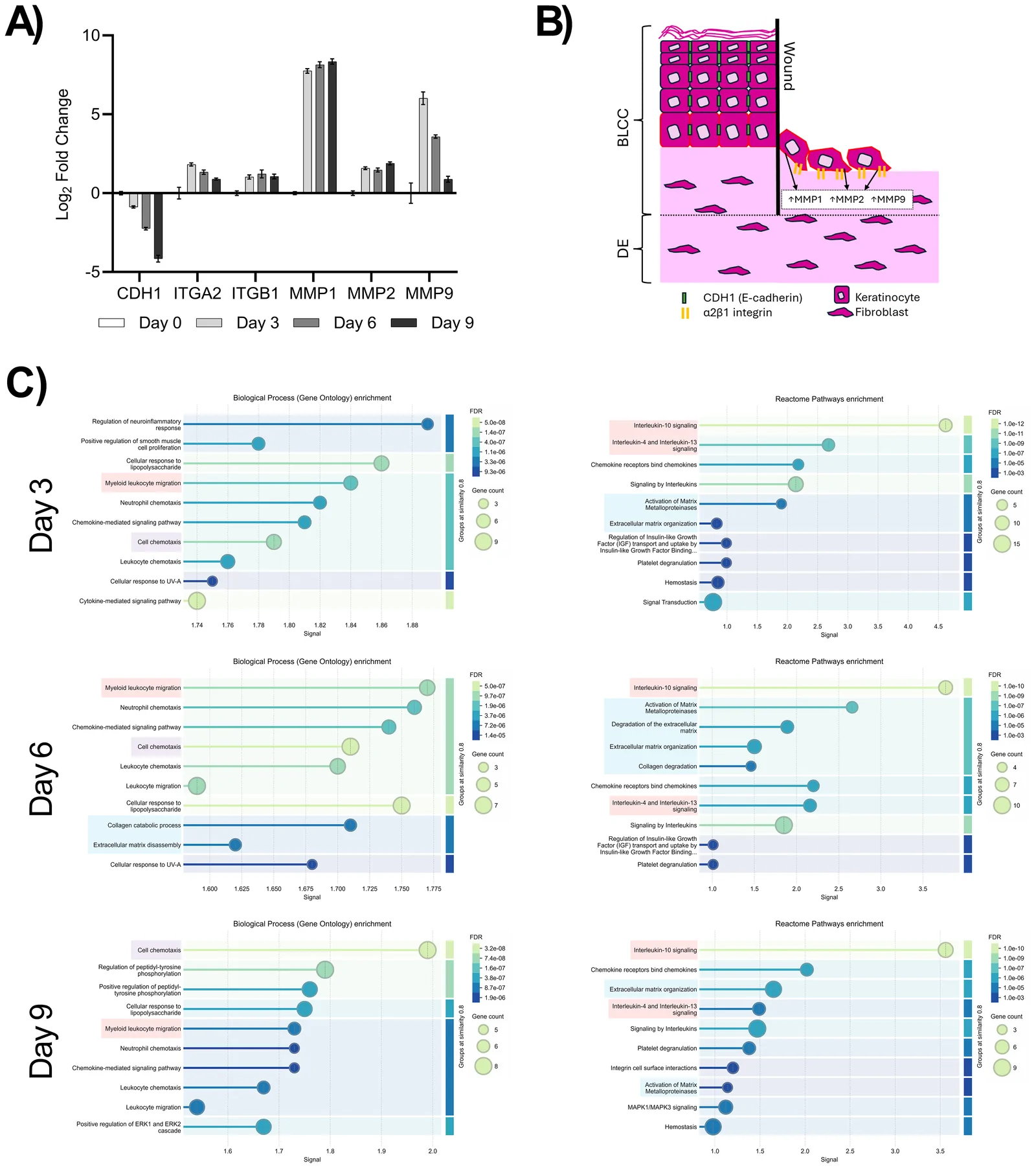
Genetic regulation promotes pathways associated with healing and immune recruitment. **(A)** Specific genes of interest from **Figure 3** involved in EMT of keratinocytes in response to wounding. Data presented as means ± standard deviations. **(B)** Cartoon schematic of keratinocyte migration during re-epithelialization highlighting genes from **(A)**. **(C)** Gene ontology and reactome pathway assessment. Red boxes=myeloid pathways, blue boxes=remodeling pathways, and purple boxes=cell chemotaxis. EMT, epithelial-to-mesenchymal transition; BLCC, bi-layered living cellular construct; DE, dermal equivalent.

### BLCC conditioned media promotes monocyte recruitment

With biological processes from the GO terms indicating a strong immune cell recruitment/activation phenotype, these findings were validated by assessing whether these genetic changes resulted in protein generation as well. Three of the genes most upregulated and statistically significant on the volcano plots at all timepoints (**Figure 3B**) were C-C Motif Chemokine Ligand 7 (CCL7), Granulocyte-Macrophage Colony-Stimulating Factor (CSF2 or GM-CSF), and Granulocyte Colony-Stimulating Factor (CSF3 or G-CSF), all of which could play roles in the recruitment and activation of monocytes into M1 or M2 phenotypes (**Figure 5A**). Assessment of spent/conditioned media found that as the BLCC epithelializes, CCL7 and G-CSF levels increase, while GM-CSF has an inverse relationship (**Figure 5B**). A transwell migration system was utilized to assess for a chemotactic response of THP-1 cells, where cells were allowed to migrate in response to conditioned media (**Figure 5C**). Recruitment was observed with conditioned media from each timepoint, with a statistical increase in migrated cells over assay media observed at Day 9, which correlated with the greatest concentration of CCL7 (**Figure 5D**). Interestingly, average cell size was shown to also be statistically increased for cells exposed to conditioned media, suggesting the presence of growth factors and proteins that could polarize the cells into different phenotypes (**Figure 5E**).

**Figure 5:**
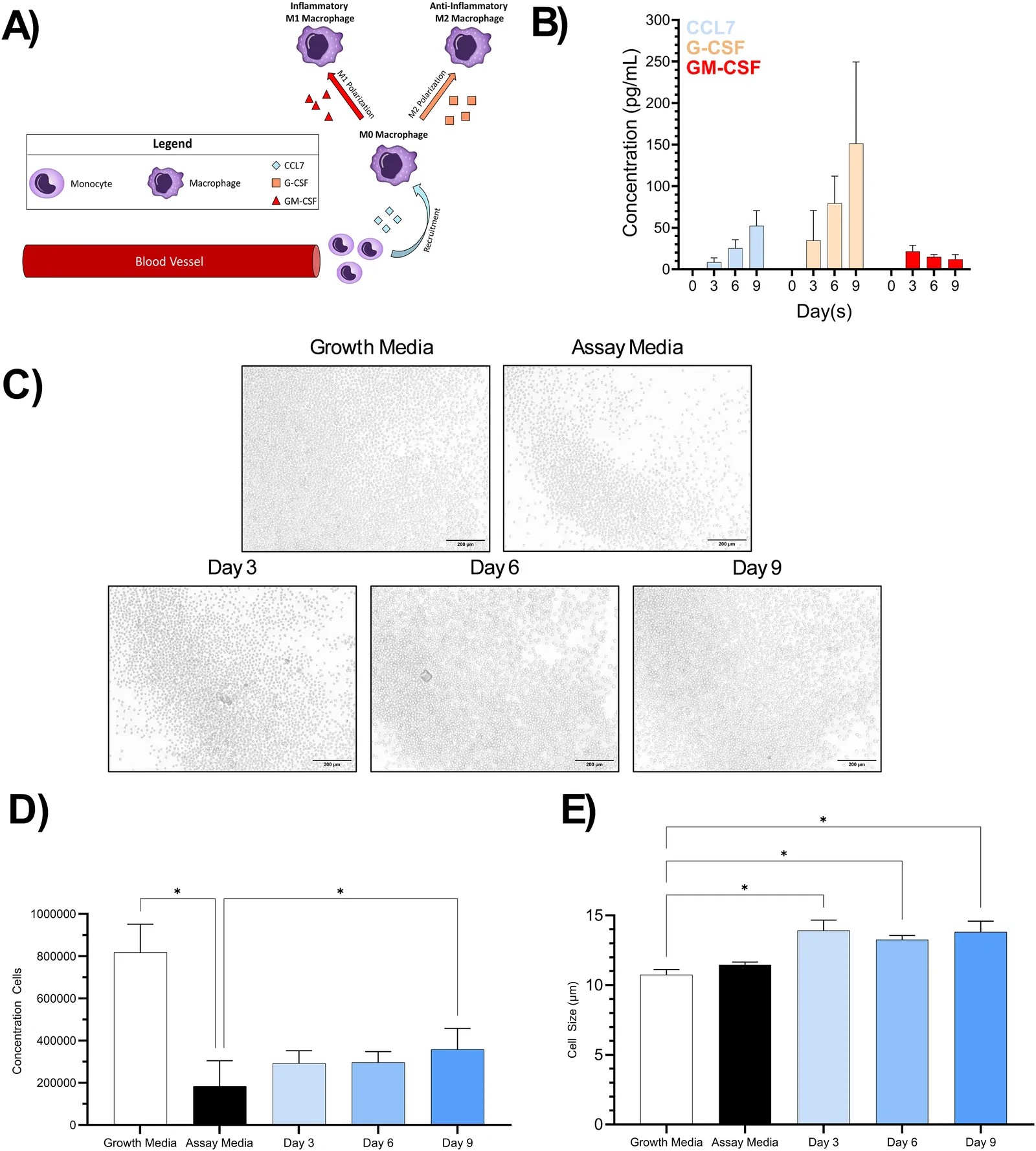
Proteins generated by BLCC wounding impact recruitment and activation of monocyte/macrophages. **(A)** Model of proteins’ impact on immune microenvironment. **(B)** Protein expression measured by ELISA related to recruitment of monocytes (CCL7), M2 polarization (G-CSF/CSF2), and M1 polarization (GM-CSF/CSF3). N = 4–6 assessed per timepoint/protein. Data presented as means ± standard deviations. **(C)** Representative image of THP-1 cells that migrated through the transwell in response to growth media (10% FBS in RPMI1640), assay media (2.5% FBS in RPMI1640), or conditioned media (50% conditioned media in 5% FBS in RPMI1640). Scale bar set to 200μm. **(D)** Cell counts from media in wells. N = 3 for controls, N = 6 for conditioned media samples. **(E)** Average size of cells that migrated in response to stimulus. N = 3 for controls, N = 6 for conditioned media samples. One-way ANOVA with Dunnett multiple comparisons; *p ≤ 0.05. CCL7, Monocyte Chemotactic Protein-3; G-CSF, Granulocyte Colony-Stimulating Factor; GM-CSF, Granulocyte-Macrophage Colony-Stimulating Factor.

### Recruited monocytes are preferentially driven towards an M2 phenotype

To assess whether conditioned media from wound healing BLCCs can promote an M2-like phenotype, surface markers indicative of M1 or M2 phenotypes on PMA stimulated THP-1 cells (M0 macrophages) exposed to spent/conditioned media were assessed (**Figure 6**). For M1 macrophages, CD80 and CD86 expression was evaluated (**Figure 6A**; **Supplementary Figure 1A**); regardless of timepoint, macrophages resembled an M2 response, with LPS and IFN-γ having a uniquely positive M1 expression profile (**Figure 6B**). For M2 macrophages, CD163 and CD206 were used as defining markers of interest (**Figure 6A**; **Supplementary Figure 1B**). While trending toward an M2-like phenotype, there was no statistical difference between groups at 24 hours; however, after 48 hours of stimulation, a statistical difference between IL-4/IL-13 stimulated cells as well as cells stimulated with spent/conditioned media compared to LPS/IFN-γ stimulated cells was observed (**Figure 6C**).

**Figure 6:**
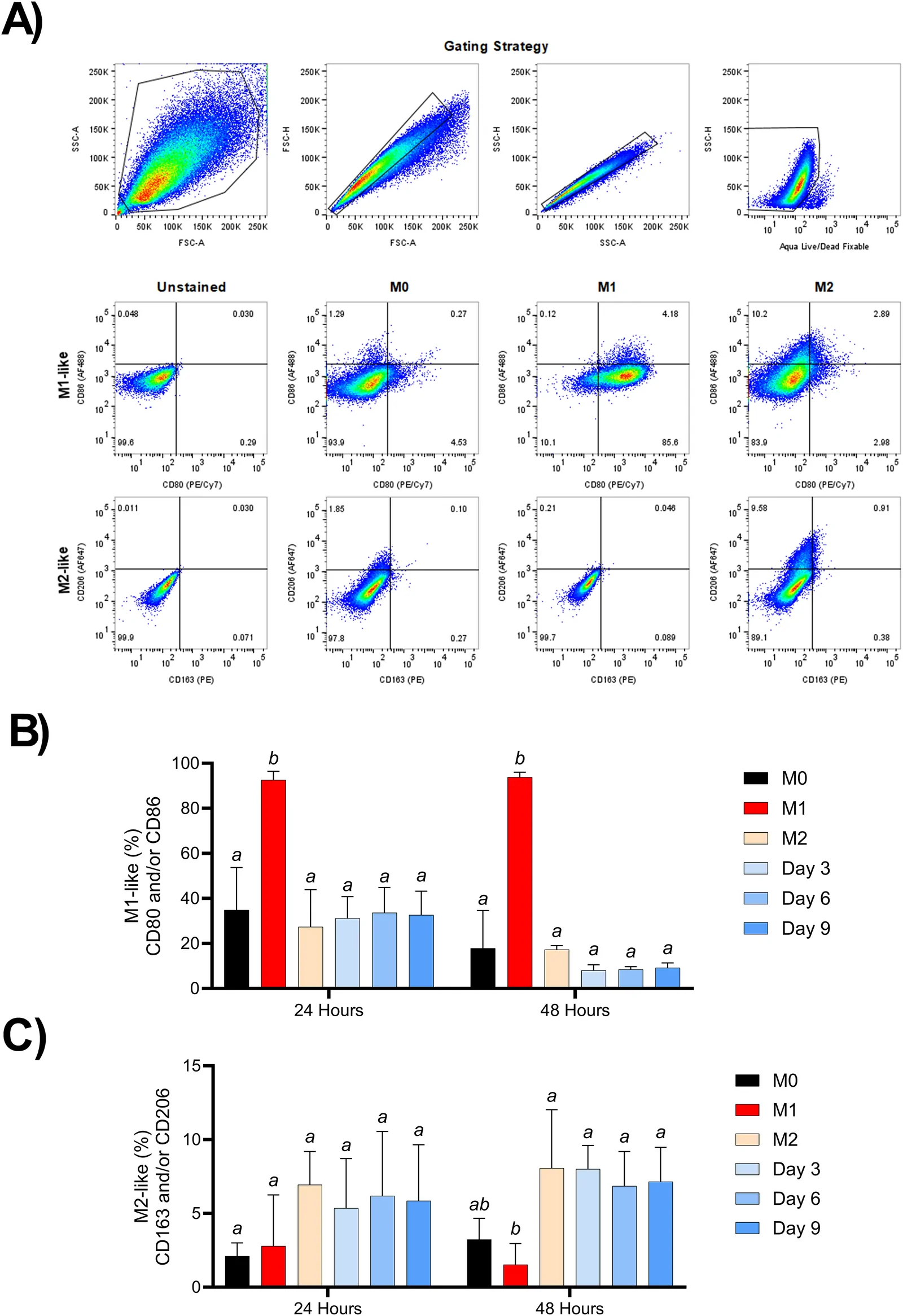
Conditioned media from healing BLCC promotes an M2-like phenotype. **(A)** Gating strategy. M1-like were defined as CD80+ and/or CD86 +. M2-like were defined as CD163+ and/or CD206 +. **(B)** Percentage of cells that were M1-like after stimulation with defined cytokine cocktail or 25% spent/conditioned media. N = 2 for controls, N = 4 for spent/conditioned media samples. **(C)** Percentage of cells that were M2-like after stimulation with defined cytokine cocktail or spent/conditioned media. N = 2 for controls, N = 4 for spent/conditioned media samples. Two-way ANOVA with Fisher’s LSD test, compact letter display statistics; p ≤ 0.05. FSC, forward scatter; SSC, side scatter; PE, phycoerythrin; AF488, Alexa Fluor 488; PE-Cy7, phycoerythrin-cyanine7; AF647, Alexa Fluor 647; CD, cluster of differentiation.

## Discussion

This study highlights how *in vitro* BLCC, during a healing response, modulates the surrounding immune microenvironment, promoting an anti-inflammatory wound healing response. Through a combination of genomic assessment, protein composition characterization, and *in vitro* models for recruitment and activation, BLCC was shown to recruit monocytes to the wound bed and provide polarization signals to drive an M2-like phenotype in a monocyte cell line.

Unlike traditional wound dressings, like gauze or collagen sheets, BLCC actively promotes healing of the wounds through various mechanisms of action. BLCC is a living, viable CTP containing a range of extracellular matrix components, growth factors, and cytokines as highlighted in **Figure 2**. Because it is made of living cells, BLCC has shown to mount a response to changes in its environment like wounding, resulting in genetic shifts and generation of various cytokines and growth factors. Previous studies by Falanga found that IL-6 was elevated out to 6 days post injury, helping to facilitate the shift from first line neutrophil inflammatory response to monocyte/macrophage recruitment (13, 23). This work expands on this concept, demonstrating how genetic regulation shifts throughout the BLCC wound healing process impact the *in vitro* wound healing process in **Figure 3**.

The BLCC wound healing process is largely driven by the expansion and migration of keratinocytes across the dermal equivalent layer. As such, genes involved in the EMT of keratinocytes in the *in vitro* wound healing process were highlighted in **Figures 4A and B**. When looking at the matrix metalloproteinases (MMPs), this study showed similar findings as those from cutaneous wounds, with upregulation of MMP1 after activation of integrin α2β1 with collagen I and the time dependent response of MMP9, with elevated expression during early phases that drops off prior to maturation of the newly formed epidermis (24). Complex genetic pathways control the genetic switch, such as the regulation of E-cadherin and N-cadherin, which are responsible for cell-cell junctions traditionally found in epithelial cells (25). Furthermore, ECM remodeling and interactions of the cells with the ECM has an impact on EMT, with increased expression of integrin α2β1 and its interactions with collagen type I resulting in disruption of E-cadherin complexes (26).

*In vivo*, wound healing is a delicate balance of inflammation and anti-inflammatory responses throughout four phases, with early phases involving elevated levels of inflammation to clear the wound of infection and bioburden and the later phases meant to resolve the inflammation and promote proliferation and remodeling of the wound bed (27). Dysregulation in this process results in chronic wounds that are stalled out in the inflammatory phase, with persistent recruitment and activation of neutrophils and M1-polarized macrophages (28). For diabetic foot ulcers (DFUs), dysregulation of the immune system and chronic hyperglycemia results in a stall during the inflammatory phase, preventing the wound from transitioning to the proliferative phase of wound healing (29). Similarly, venous leg ulcers (VLUs) are resultant of exacerbated inflammation driven by venous hypertension, resulting in the extravasation of inflammatory cells and subsequent release of cytokines and proteases (30). In mechanism of action studies utilizing BLCC-treated VLUs, it was found that treatment with BLCC resulted in a shift from chronic inflammation toward an acute, pro-healing wounding response, with gene ontology indicating a role for leukocyte chemotaxis and activation (8, 9). In line with these important findings, promotion of an M2 phenotype, as demonstrated in **Figure 6**, could therefore help shift the immune microenvironment to a pro-healing proliferative state.

Some key limitations within this study are a focus on monocyte/macrophage recruitment and activation. CCL7 is known to attract several leukocytes, including neutrophils and T cells, both of which can play a role in modulating the inflammatory and wound healing environments (31). Future studies focused on additional immune cell populations and how signals from BLCC could impact their recruitment and activation are warranted. While THP-1 cells are a well-utilized immortalized line for addressing the role of macrophages *in vitro* (32), they may not fully recapitulate the *in vivo* response seen in humans (33). Finally, the addition of a larger genetic or proteomic panel could highlight additional means with which BLCC could modulate the immune system outside of traditional wound healing targets.

In conclusion, BLCC not only was shown to promote wound healing of the product itself when wounded but can also support an anti-inflammatory wound healing environment *in vitro*. This wound healing response is resultant of genetic shifts and protein generation that promotes the re-epithelialization of the product and subsequent recruitment and activation of monocyte/macrophages. Spent/conditioned media from wounded BLCC promoted chemotaxis of THP-1 cells and subsequent M2-like polarization, a critical immune cell effector for the promotion of a wound healing phenotype. These findings help to further characterize the mechanism of action with which BLCC, a Premarket Approval CTP, may support the wound healing environment.

## Data Availability

The original contributions presented in the study are included in the article/**Supplementary Material**; further inquiries can be directed to the corresponding author.

## Supplementary material

Supplementary Figure 1 Representative gates from BLCC treated samples promoting an M2-like phenotype. **(A)** Representative flow plots showing M1-like status after 24- and 48-hours of stimulation with defined cytokine cocktail or 25% spent/conditioned media. **(B)** Representative flow plots showing M2-like status after 24- and 48-hours of stimulation with defined cytokine cocktail or 25% spent/conditioned media. PE, phycoerythrin; AF488, Alexa Fluor 488; PE-Cy7, phycoerythrin-cyanine7; AF647, Alexa Fluor 647; CD, cluster of differentiation.

Supplementary Table 1 Antibodies used in immunofluorescence staining.
